# Genome-Wide Epistatic Interaction Networks Affecting Feed Efficiency in Duroc and Landrace Pigs

**DOI:** 10.3389/fgene.2020.00121

**Published:** 2020-02-28

**Authors:** Priyanka Banerjee, Victor Adriano Okstoft Carmelo, Haja N. Kadarmideen

**Affiliations:** Quantitative Genomics, Bioinformatics and Computational Biology Group, Department of Applied Mathematics and Computer Science, Technical University of Denmark, Kongens Lyngby, Denmark

**Keywords:** epistasis, WISH, WGCNA, feed efficiency, pigs

## Abstract

Interactions among genomic loci have often been overlooked in genome-wide association studies, revealing the combinatorial effects of variants on phenotype or disease manifestation. Unexplained genetic variance, interactions among causal genes of small effects, and biological pathways could be identified using a network biology approach. The main objective of this study was to determine the genome-wide epistatic variants affecting feed efficiency traits [feed conversion ratio (FCR) and residual feed intake (RFI)] based on weighted interaction SNP hub (WISH-R) method. Herein, we detected highly interconnected epistatic SNP modules, pathways, and potential biomarkers for the FCR and RFI in Duroc and Landrace purebreds considering the whole population, and separately for low and high feed efficient groups. Highly interacting SNP modules in Duroc (1,247 SNPs) and Landrace (1,215 SNPs) across the population and for low feed efficient (Duroc—80 SNPs, Landrace—146 SNPs) and high feed efficient group (Duroc—198 SNPs, Landrace—232 SNPs) for FCR and RFI were identified. Gene and pathway analyses identified *ABL1*, *MAP3K4*, *MAP3K5*, *SEMA6A*, *KITLG*, and *KAT2B* from chromosomes 1, 2, 5, and 13 underlying ErbB, Ras, Rap1, thyroid hormone, axon guidance pathways in Duroc. *GABBR2*, *GNA12,* and *PRKCG* genes from chromosomes 1, 3, and 6 pointed towards thyroid hormone, cGMP-PKG and cAMP pathways in Landrace. From Duroc low feed efficient group, the *TPK1* gene was found involved with thiamine metabolism, whereas *PARD6G*, *DLG2, CRB1* were involved with the hippo signaling pathway in high feed efficient group. *PLOD1* and *SETD7* genes were involved with lysine degradation in low feed efficient group in Landrace, while high feed efficient group pointed to genes underpinning valine, leucine, isoleucine degradation, and fatty acid elongation. Some SNPs and genes identified are known for their association with feed efficiency, others are novel and potentially provide new avenues for further research. Further validation of epistatic SNPs and genes identified here in a larger cohort would help to establish a framework for modelling epistatic variance in future methods of genomic prediction, increasing the accuracy of estimated genetic merit for FE and helping the pig breeding industry.

## Introduction

Genetic or epistatic interactions between SNPs, genes or QTLs is a topic of interest in molecular and quantitative genetics (Cordell, 2002) as it occurs when the phenotypic effect of a mutation is affected by the presence of other mutations in the genome (Papp and Pál, 2011). The study of epistasis has fascinated biologists, as these interactions are key to understand how genes relate functionally. With the knowledge gained from genome-wide association studies (GWAS), an unbiased survey of single nucleotide polymorphisms (SNPs) across the genome assayed by commercial SNP platforms are tested for association with a phenotypic trait of interest. Although most of these SNPs are significantly associated, they have shown a small effect and account for a small proportion of heritable variance (Frazer et al., 2009; Manolio et al., 2009; Eichler et al., 2010).

Epistatic interactions between genes for some quantitative traits, such as meat quality (Ovilo et al., 2002; Duthie et al., 2011a), carcass (Duthie et al., 2010), reproductive traits (Bidanel, 1993; Rodríguez et al., 2005; Noguera et al., 2009), growth (Crooks and Guo, 2017), and muscle fiber traits (Estellé et al., 2008) have been reported for pigs. These findings shed light on the different interactions underlying the genomic regulation in several traits. Although, a large number of GWAS studies for feed efficiency and feeding behavior traits in pigs have been reported (Ding et al., 2017; Reyer et al., 2017b; Ding et al., 2018; Zhang et al., 2018), the epistatic effects over these traits are still not explored. By using 88 microsatellite markers in pigs, (Duthie et al., 2011b) epistatic QTLs (Quantitative Trait Loci) for growth, feed intake and chemical body composition at different stages of growth have been reported. However, to the best of our knowledge, there are no studies on genome-wide epistatic interactions using high throughput genomic data (HTG) and in the context of genetic (SNP) networks underlying feed efficiency related traits in pigs.

GWAS being a single-step approach tests each SNP with the trait of interest, which gives rise to multiple testing problems. Additionally, due to the high stringent cutoffs, some of the most biologically relevant SNPs are missed. Thus, searching for the missing heritability of the traits has led us to include the genetic or epistatic interactions, as it would help us to identify markers explaining a higher proportion of heritable variance. Furthermore, if all the epistatic SNPs are identified, it would help to establish a framework to explicitly include epistatic variance in future methods of genomic prediction in addition to additive variance. This approach would increase the accuracy of genomic prediction for FE and help the pig breeding industry.

Systems genomics and network biology methods focus on detecting interactions among genes and relate them to phenotype or disease manifestation (Suravajhala et al., 2016). WISH (Weighted Interaction SNP Hub) network method (Kogelman and Kadarmideen, 2014) has been proposed to calculate genome-wide epistatic interactions and to construct epistatic networks underlying complex phenotypes. Recently implemented in R, as WISH-R package (Carmelo et al., 2018), this method detects genome-wide interactions between single nucleotide polymorphism (SNP) and traits in high throughput genomic data (HTG). WISH-R calculates epistasis and constructs biological networks using Weighted Gene Co-expression Network Analysis (WGCNA) framework (Langfelder and Horvath, 2008). Herein, the main assumption of WGCNA for clustering gene expression data is used for HTG data to develop a scale-free weighted genetic interaction network to detect biologically relevant genetic modules and biological pathways for complex traits. Herein, we carried out a genomic scan for epistatic interaction followed by network analyses to investigate the extent of epistatic interactions underlying feed efficiency traits in Duroc and Landrace pigs. Additionally, we reported the biological pathways between both the breeds and within efficient and inefficient animals.

## Material and Methods

### Study Design and Phenotypes

**Figure 1:**
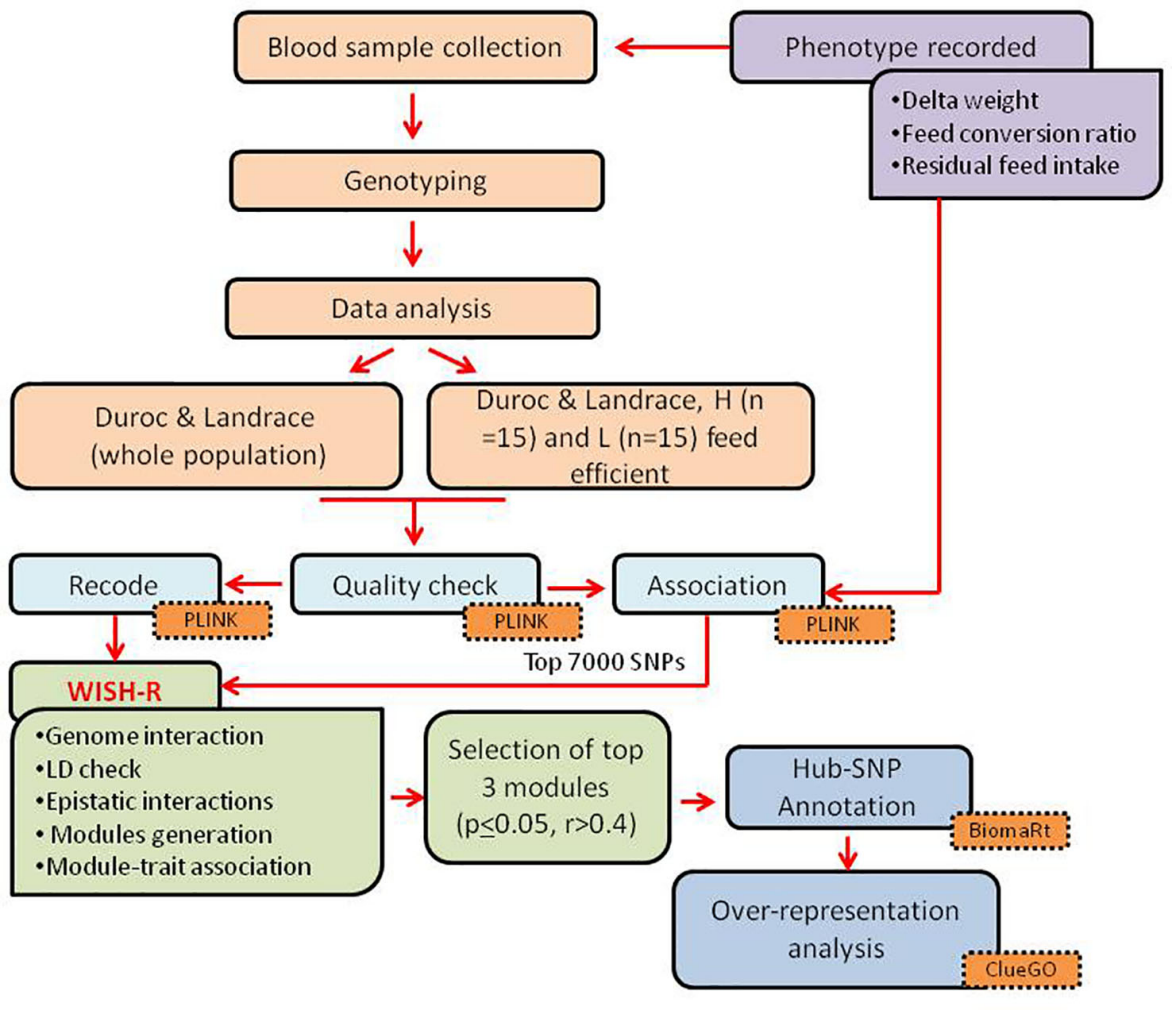
Schematic representation of the study design and analyses steps.

A general overview of the study design and the main analyses steps is represented in **Figure 1**. The experimental trial was conducted at the pig testing station “Bøgildgård” operated by SEGES within Landbrug and Fødevarer (L&F: Danish Agriculture and Food Council). Pigs were *ad libitum* fed and free water supplied. The authors of this study were not responsible for animal husbandry, diet, and care as the testing station is a facility within the Danish breeding program run by SEGES.

Blood samples at Bøgildgård were collected from jugular venipuncture from each pig into tubes containing EDTA and immediately placed on ice. The pigs were purebred uncastrated males from Danbred Duroc (n = 59) and Danbred Landrace breeds (n = 50), amounting to a total of 109 animals. The breeds have been mentioned as Duroc and Landrace further in the paper.

For the phenotypic traits, the weight of feed consumed (FC) and FE for each pig in the testing phase were measured, beginning with an initial weight of around 28 kg for each animal. The testing phase ranged from 41 to 70 days based on the viability of each pig. Bodyweight during the beginning and end of the test were recorded from the standard test procedure of the testing station, and their difference was referred to as delta weight (DW). The feed conversion ratio (FCR) was calculated as the ratio between the amount of FC and DW. Likewise, the residual feed intake (RFI) was estimated as the difference between the observed daily feed intake (DFI) and the predicted feed intake (pDFI) (Nguyen and McPhee, 2001). A summary of the traits is shown in **Table 1**. The animals were also classified as low and high feed efficient groups (LFE and HFE, respectively), based on the extremities of FCR for each breed. This was done by selecting pigs that were one standard deviation above or below the mean FCR for each breed, resulting in 15 samples in the LFE and HFE groups. The in-group analysis was done using RFI and FCR as a continuous trait.

**Table 1:** Descriptive statistics of the phenotypic traits from Duroc and Landrace evaluated for feed efficiency related-traits.

Parameter	Samples	Feed consumed*	Delta weight*	Feed conversion ratio*	Age*^,#^
Abbreviations	N	FC	DW	FCR	
Unit		kg/day	kg		days
Duroc	59	128.22 ± 16.84	65.08 ± 8.08	1.96 ± 0.07	125.12 ± 3.12
DLFE	15	134.93 ± 17.83	65.13 ± 8.98	2.07 ± 0.05	126.4 ± 2.72
DHFE	15	121.13 ± 20.41	64.33 ± 10.37	1.88 ± 0.03	124.8 ± 3.0
Landrace	50	133.54 ± 23.81	63.36 ± 10.93	2.11 ± 0.08	136.94 ± 3.18
LLFE	15	144.87 ± 20.95	65.8 ± 9.28	2.32 ± 0.09	137.8 ± 3.25
LHFE	15	131.47 ± 26.48	65.13 ± 12.8	2.13 ± 0.09	136.06 ± 2.19

*Values represent mean ± standard deviation, ^#^Age at the end of the testing period. DLFE, Duroc low feed efficiency; DHFE, Duroc high feed efficiency; LLFE, Landrace low feed efficiency; LHFE, Landrace high feed efficiency.

### SNP Genotyping, Quality Control, and Association

DNA isolation was carried out from the collected blood, and SNP genotyping was outsourced to GeneSeek (Neogen company - https://www.neogen.com/uk/). Genotyping was conducted using GGP Porcine HD array (GeneSeek, Scotland, UK), featuring over 68,516 SNPs across 18 autosomes and two sex chromosomes.

The genotype quality control (QC) was carried out using Plink software (Purcell et al., 2007). Samples with a genotyping rate ≤ 90% of markers were filtered out. SNPs with call rates < 90%, minor allele frequencies < 0.01, and Hardy-Weinberg equilibrium (HWE) P < 1 × 10^-7^, and the SNPs located on sex chromosomes, as well as with no position information were also excluded from the dataset.

To identify meaningful SNPs related to FCR, both among and within breeds (LFE and HFE groups), we carried an association analysis using Plink version 1.07 based on the “*assoc*” function. For this, two approaches were adopted as follows: we first carried a breed-specific analysis, considering the whole population (Duroc—59 samples and Landrace—50 samples). Then, the association was also performed within groups for each breed to identify group specific SNPs. The computed p-values were used in the later steps as a filter.

### Genome-Wide Pair-Wise Interaction Analysis

We carried out a genome-wide association to investigate the epistatic interactions for feed efficiency traits in Duroc and Landrace pigs by applying the WISH-R package (Carmelo et al., 2018) based on WISH method (Kogelman and Kadarmideen, 2014). To identify breed-specific and group-specific SNP interactions, we selected the top 7,000 SNPs associated to FCR from each analysis, and then carried out the epistatic interaction calculation individually for each breed and each group (LFE, HFE). The selected SNPs were pruned for linkage disequilibrium (LD) using “*LD_blocks*” function, with a maximum block size of 1000, and threshold = 0.9 from WISH-R package (Carmelo et al., 2018). Further, the “*epistatic.correlation*” function, considering the default parameters, was employed to calculate the epistatic interaction among the remaining SNPs (Kogelman and Kadarmideen, 2014; Carmelo et al., 2018). The FCR was used as a continuous trait in the analyses for LFE and HFE groups and in both the breeds.

The heterogeneity model used for calculating epistasis is given as:

$$y=\text{μ}+\beta_{1}{snp}_{i}+\beta_{2}{snp}_{j}+\beta_{3}({snp}_{i}\;x\;{snp}_{j})+\text{ε}$$

where y is the phenotype of interest, µ is the intercept, *β_1_* and *β_2_* are the SNP main effects, *ϵ* is the random residual effect, *β_3_* represents the epistasis of the two loci. To represent the genotype *snp_i_* and *snp_j_*, the genotype data were coded as 2 (homozygote minor alleles), 1 (heterozygote) or 0 (homozygote major alleles) based on the function “*generate.genotype*” in WISH-R which uses file in the PED and TPED file format from Plink (Purcell et al., 2007).

The epistatic interactions (*β_3_*) estimated by WISH-R were visualized using the function “*genome.interaction*” with a quantile size of 0.9. The genome-wide interaction overview was calculated with the quantile values of the significance of the interaction between chromosomes. It indicated the chromosomal hotspots for the interaction of a given phenotype. WISH-R default settings were used to calculate chromosomal interactions.

### Epistatic Networks and Trait Association Analyses

The SNP-SNP epistatic correlation coefficients calculated were used to construct the epistatic networks following the WISH-R pipeline that identified modules of SNPs related by epistasis. We then summarized modules based on module eigenSNP (MSNP), and related the MSNP with the traits of interest. We constructed a signed weighted network, using “*generate.module*” function, separately for both breeds, and also for the LFE and HFE groups in each breed. Strongly correlated modules were merged using the function “*mergeCloseModules”*.

To unravel biologically meaningful modules, we used the module eigenSNP to examine the relationship between the FCR and the modules, resulting in a genome-wide module association test. We also included the RFI in the association analysis to identify epistatic SNPs for both the traits.

The top 3 significant modules (p ≤ 0.05 and r > 0.4) were selected for downstream analysis to unravel the biological pathways underlying the traits. The P-values used were adjusted using Bonferroni approach referred as *P_adj_*. The highest interacting SNPs were identified considering the SNP connectivity score estimated from the “*softConnectivity*” function (Langfelder and Horvath, 2008). The SNPs from the top significant modules were termed as hub-SNPs and selected for further analysis.

### Annotation of SNP-SNP Network and Gene Identification

The SNP annotation was done using BiomaRt (R package) (Durinck et al., 2005; Durinck et al., 2009) from the Porcine *Ensembl* database, *Sscrofa11.1*. The unidentified SNP annotation from Biomart was done through g:Convert in g:Profiler (https://biit.cs.ut.ee/gprofiler/convert) (Reimand et al., 2007). Gene ontologies (GO), canonical pathways, and biological interpretation were performed from ClueGO version 2.5.4 (Cytoscape plugin) (Bindea et al., 2009) based on *Sus scrofa* annotation. The KEGG pathways over-represented in the selected modules were identified by grouping the redundant terms with kappa-score = 0.4. The pathways with GroupPValue ≤ 0.05 were selected, and the network was visualized using Cytoscape version 3.7.1 (https://cytoscape.org/) (Shannon et al., 2003). A heatmap was constructed using pheatmap R-package considering -log10 (GroupPvalue) to summarize the pathways within and among the groups.

## Results

### Genome-Wide Epistatic Interactions

After quality control (QC), 48,444 and 49,876 loci in Duroc and Landrace on 18 porcine autosomes were available for association analysis with FCR. Likewise, 51,852 and 51,335 SNPs from Duroc and Landrace, respectively, passed QC in LFE and HFE groups.

**Figure 2:**
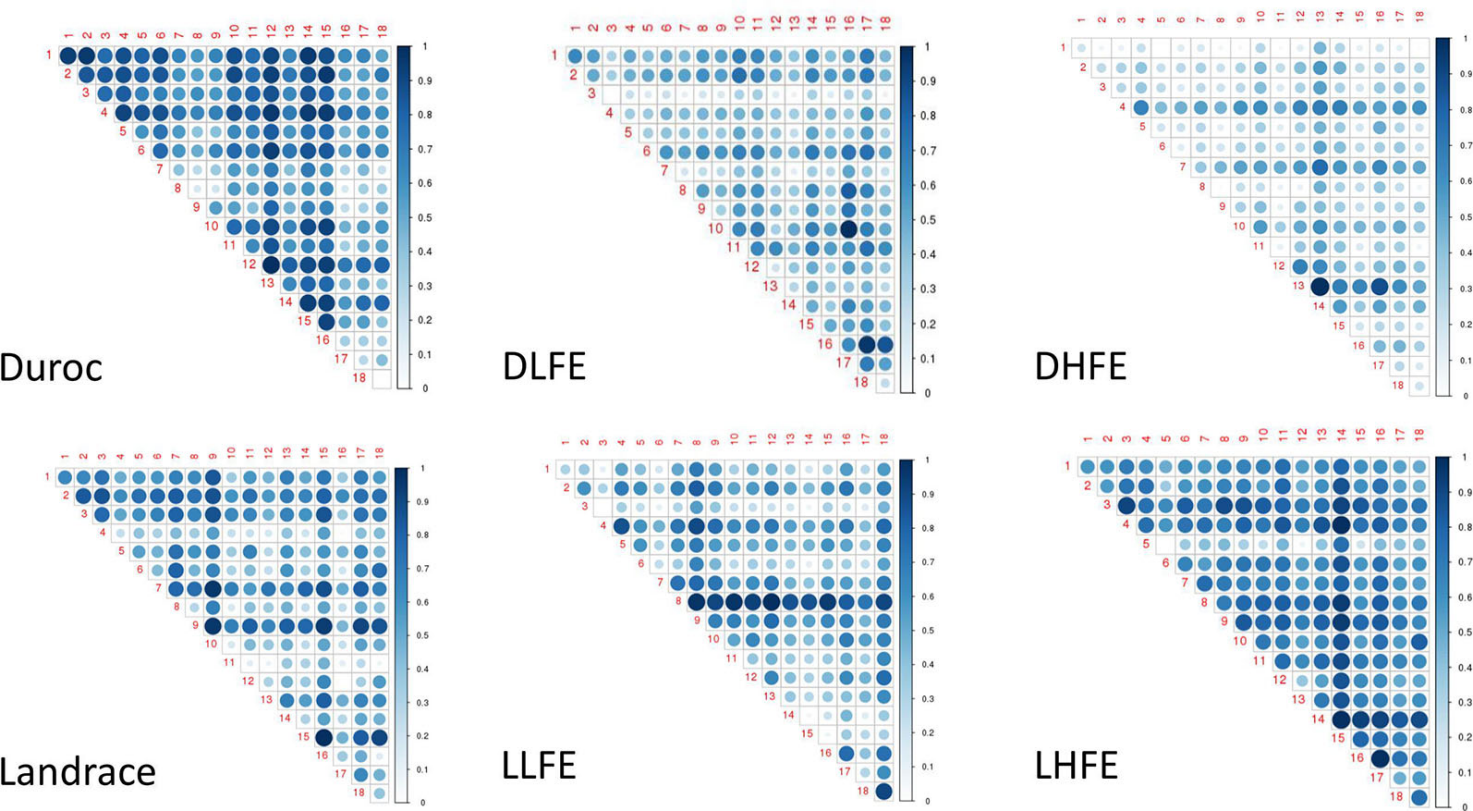
Pairwise chromosomal interaction. DLFE and DHFE represent Duroc low and high feed efficient groups, LLFE and LHFE represent Landrace low and high feed efficient groups, respectively.

From the association analyses, the top 7,000 SNPs were selected, and based on WISH-R package (Carmelo et al., 2018), we calculated the epistatic interactions based on FCR in both the breeds and within the breed groups (**Figure 2**). In Duroc considering the whole population, the chromosomal hotspots were widely distributed. However, in the Duroc, low feed efficient (DLFE) group, the pairwise interaction was more evident in chromosomes 10, 16, and 17 (**Figure 2**). From the Duroc, high feed efficient (DHFE) group (**Figure 2**), pairwise interactions were evident in chromosomes 4, 7, and 13. Although a widespread pairwise interaction was seen considering the whole population of the Landrace (**Figure 2**), the pairwise interaction in low feed efficient (LLFE) group was evident only in chromosomes 8 and 18 (**Figure 2**) whereas the chromosome 14 stood out for high feed efficiency (LHFE) group (**Figure 2**).

### SNP-SNP Network Analysis

**Figure 3:**
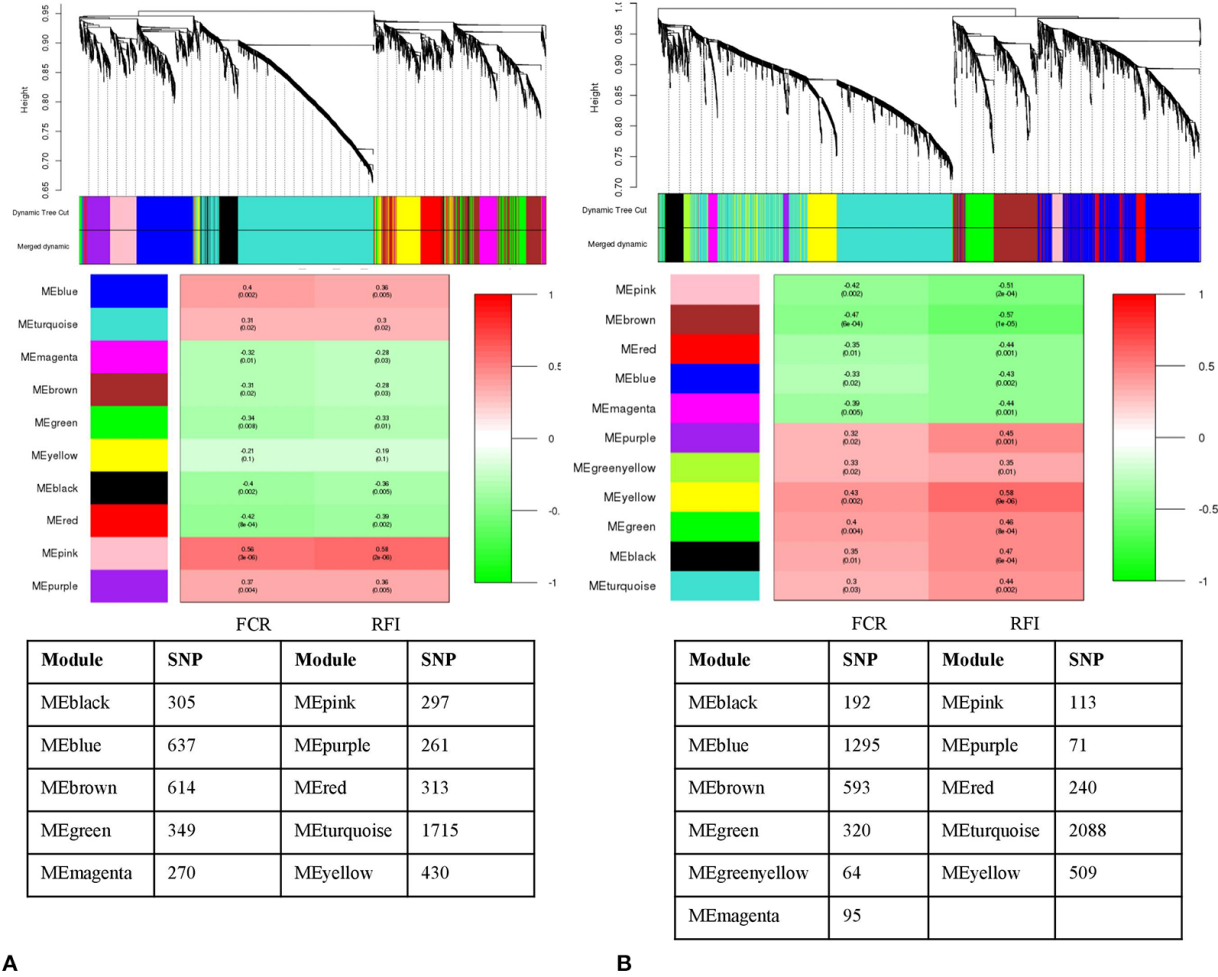
Dendrogram and module-trait correlation plots in A: Duroc and B: Landrace, with FCR and RFI as a continuous trait. Upper panel of each plot **(A, B)** represents SNP-clustering dendrogram obtained by hierarchical clustering of TOM-based dissimilarity with the corresponding module colors indicated by the color row. Each colored row represents color-coded module that contains a group of highly connected SNPs. The middle panel of each plot **(A, B)** represents the module trait correlation, where the x-axis represents feed conversion ratio (FCR) in the first column and residual feed intake (RFI) in the second column; the y-axis represents the modules. The color-code in the module-trait correlation plots is based on Pearson’s correlation (p-values in parenthesis). Positive and negative correlations are shown in red and green colors, respectively. The lower panel represents the number of SNPs clustered in each module.

Clustering the 5,191 remaining SNPs after LD pruning, we identified 10 modules in Duroc. Based on our criteria of r >0.4 and p value ≤ 0.05, three modules were selected for further analysis. Among the module-traits, MEpink (297 SNPs), MEblue (637 SNPs) and MEred (313 SNPs) were significantly associated (P*_adj_* <0.01) with both FCR and RFI traits (**Figure 3A**). For Landrace, 5,580 SNPs were clustered into 11 modules after LD pruning. Among the identified modules, MEbrown (593 SNPs), MEpink (113 SNPs), and MEyellow (509 SNPs) were the top significant ones associated to FCR and RFI. (**Figure 3B**). The list of hub-SNPs and their connectivity score is given in **Supplementary Table 1**.

**Figure 4:**
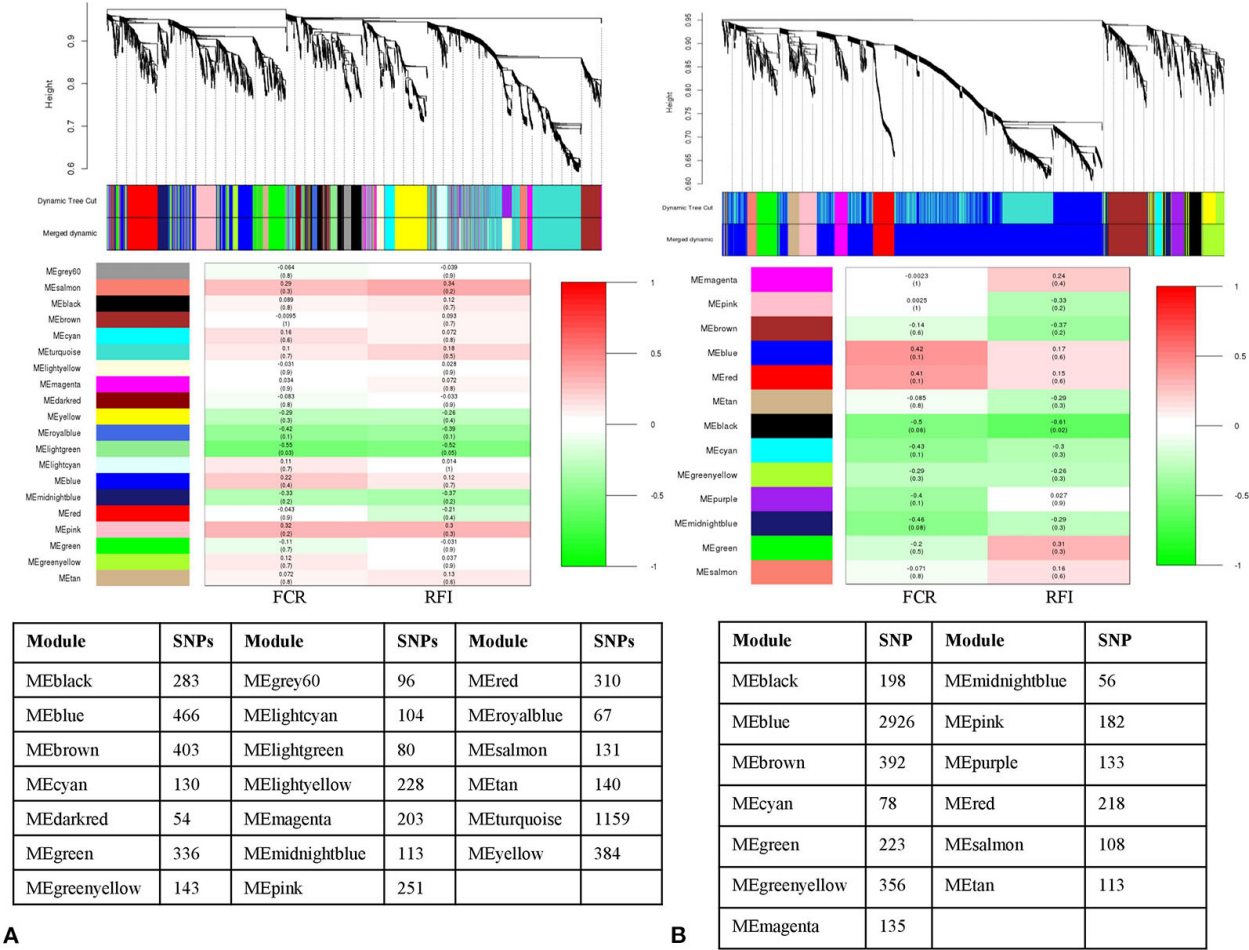
Dendrogram and module-trait correlation plots in A: Duroc low and B: Duroc high feed efficient groups. Upper panel of each plot **(A, B)** represents SNP-clustering dendrogram obtained by hierarchical clustering of TOM-based dissimilarity with the corresponding module colors indicated by the color row. Each colored row represents color-coded module that contains a group of highly connected SNPs. The middle panel of each plot **(A, B)** represents the module trait correlation, where the x-axis represents feed conversion ratio (FCR) in the first column and residual feed intake (RFI) in the second column; the y-axis represents the modules. The color-code in the module-trait correlation plots is based on Pearson’s correlation (p-values in parenthesis). Positive and negative correlations are shown in red and green colors, respectively. The lower panel represents the number of SNPs clustered in each module.

Likewise, the SNP-SNP interaction was calculated in LFE and HFE groups in both breeds. In DLFE group, 5,081 SNPs were tested for the interaction after LD pruning. The SNPs were clustered into 20 modules in which MElightgreen with 80 SNPs was found to be the only module significant associated with FCR (r = –0.55, p-value = 0.03) and RFI (r = –0.52, p-value = 0.05) (**Figure 4A**). Regarding DHFE group, 5,118 SNPs were tested and clustered into 13 modules. The MEblack (198 SNPs) was significantly correlated to RFI (r = –0.61, p-value = 0.02) (**Figure 4B**). There was no significant association for FCR.

**Figure 5:**
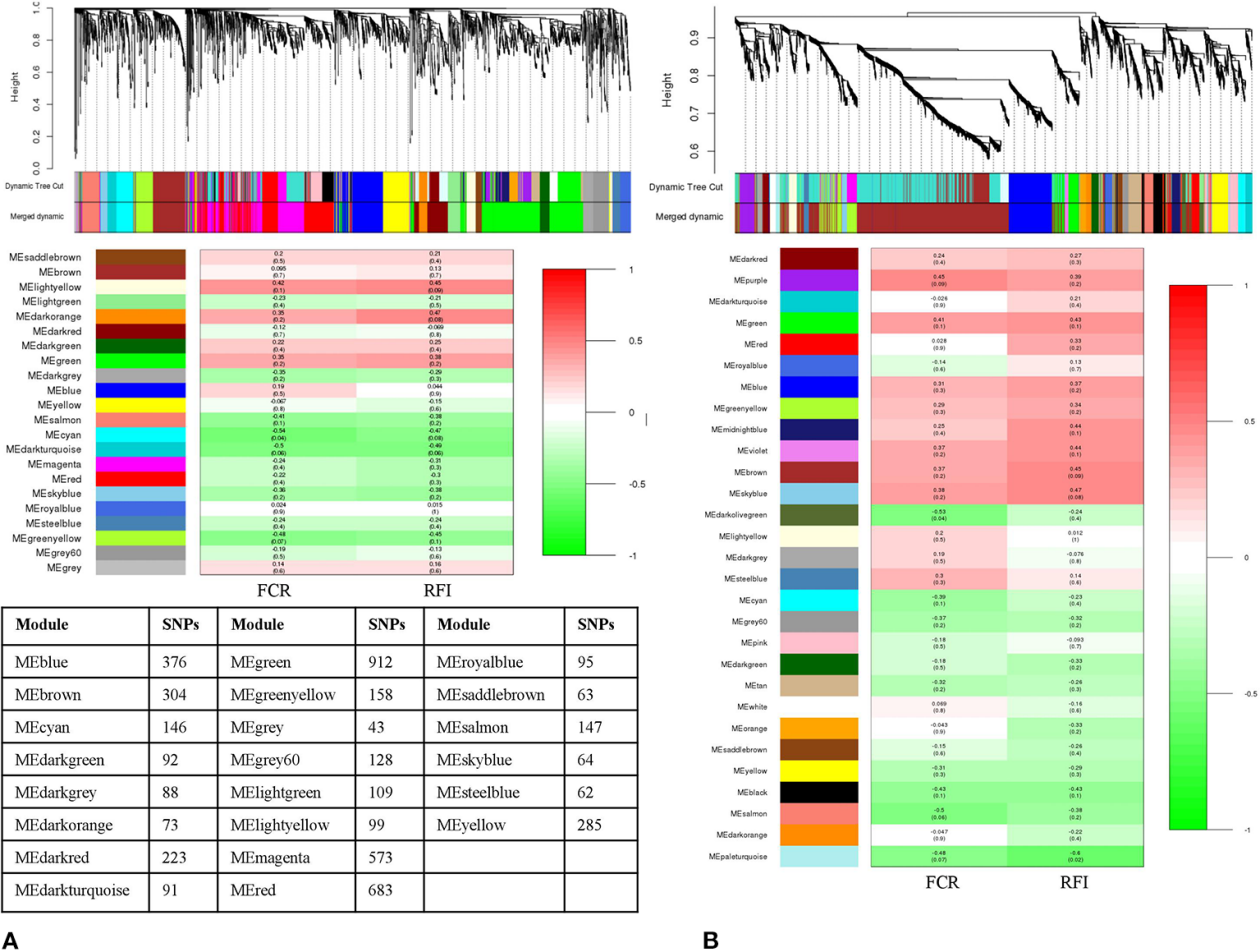
Dendrogram and module-trait correlation plots in A: Landrace low and B: Landrace high feed efficient groups. Upper panel of each plot **(A, B)** represents SNP-clustering dendrogram obtained by hierarchical clustering of TOM-based dissimilarity with the corresponding module colors indicated by the color row. Each colored row represents color-coded module that contains a group of highly connected SNPs. The middle panel of each plot **(A, B)** represents the module trait correlation, where the x-axis represents feed conversion ratio (FCR) in the first column and residual feed intake (RFI) in the second column; the y-axis represents the modules. The color-code in the module-trait correlation plots is based on Pearson’s correlation (p-values in parenthesis). Positive and negative correlations are shown in red and green colors, respectively. The lower panel represents the number of SNPs clustered in each module.

From LLFE group, 4,814 SNPs were tested and generated into 22 modules. The MEcyan module, comprising 146 SNPs (r = –0.54, p-value = 0.04) was significantly correlated with FCR. No significant associations were identified for RFI (**Figure 5A**). Among 7,547 SNPs in LHFE group, 29 modules were identified and MEdarkolivegreen (r = –0.53, p-value = 0.04) with 170 SNPs and MEpaleturquoise (r = –0.6, p-value = 0.02) with 62 SNPs were significantly correlated with FCR and RFI, respectively (**Figure 5B**).

### SNP Annotation and Network Visualization

The hub-SNPs were identified (**Supplementary Table 1**) from the significantly correlated modules (P ≤ 0.05) as described above. The summary of annotated hub-SNPs is provided in **Table 2**.

**Table 2:** Significant SNPs for FCR and RFI annotated for pathway analysis.

Breed	Number of SNPs	Module	Trait	Annotated SNPs*
Duroc	297	MEpink	FCR/RFI	686
	637	MEblue		
	313	MEred		
DLFE	80	MElightgreen	FCR/RFI	39
DHFE	198	MEblack	RFI	113
Landrace	593	MEbrown	FCR/RFI	701
	113	MEpink		
	509	MEyellow		
LLFE	146	MEcyan	FCR	69
LHFE	170	MEdarkolivegeen	FCR	85
	62	MEpaleturquoise	RFI	

DLFE, Duroc low feed efficient group; DHFE, Duroc high feed efficient group; LLFE, Landrace Low feed efficient group; LHFE, Landrace high feed efficient group; FCR, Feed conversion ratio; RFI, Residual feed intake, *Number of genes identified after annotation.

**Figure 6:**
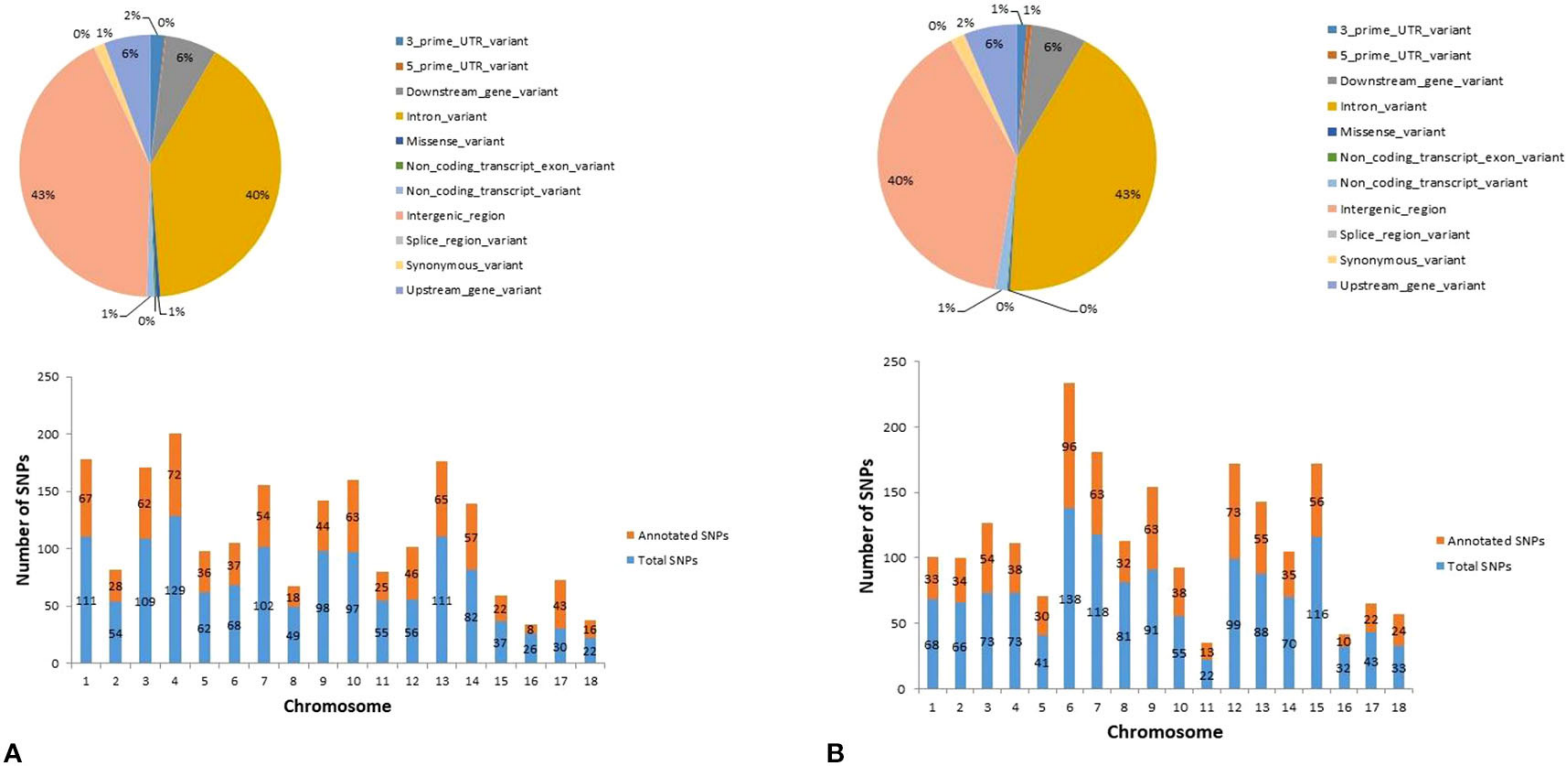
Graphical representation of the variant type identified (pie-chart) and distribution of SNPs across the chromosomes identified in the top significant modules (bar plot) for **(A)** Duroc and **(B)** Landrace.

In Duroc, out of the 1,247 hub-SNPs, 43% of the SNPs were harbored in intergenic regions, followed by 40% in intronic variants (**Figure 6A**—pie chart). Regarding Landrace, out of 1,215 hub-SNPs identified, 43% of the SNPs were intronic variants, followed by 40% of SNPs in intergenic regions (**Figure 6B**—pie chart). The distribution of SNPs across the chromosomes identified from the significant modules and number of SNPs annotated in each breed are provided in the bar plot for Duroc and Landrace, respectively (**Figures 6A, B**—bar plot).

**Figure 7:**
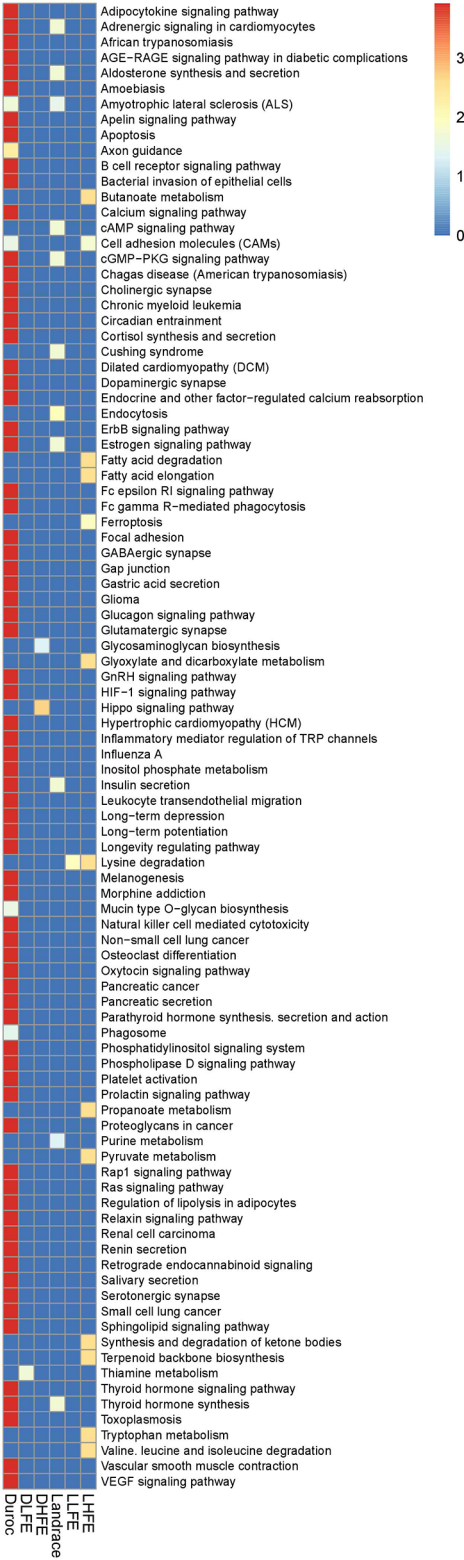
Over-represented signaling pathways of genes identified from hub-SNPs in each breed with low and high feed efficient groups. The plot is color-coded based on -log10 (GroupPvalue) of different pathways.

From Duroc (1,247 hub-SNPs) and Landrace (1,215 hub-SNPs), only 64 SNPs were found common, while 1,183 SNPs were unique to Duroc and 1,151 were unique to Landrace. In DLFE and DHFE groups, with 80 and 198 hub-SNPs, respectively, four SNPs were common pointing towards the difference in SNP interactions in both breeds. In LLFE and LHFE groups, among 146 and 232 hub-SNPs in each group, respectively, only six SNPs were found common between them. The annotated hub-SNPs (**Table 2**) corresponding to genes were subjected to pathway analysis in ClueGO to capture the biological information of each gene in all the groups. The significant pathways (GroupPValue ≤ 0.05) underlying the hub-SNPs identified for each breed and in low and high feed efficient groups are given in **Supplementary Table 2** and **Figure 7**.

The pathways identified in Duroc points to inositol phosphate metabolism, *Erb* signaling pathway, *Ras* signaling pathway, and *Rap1* signaling pathway. For DLFE group, SNP-SNP interaction pointed out to thiamine metabolism and phenylalanine, tyrosine, and tryptophan metabolism. In DHFE group, the pathways resulting from the interaction were the hippo signaling pathway and glycosaminoglycan biosynthesis. In Landrace, the major pathways involved were cGMP-PKG signaling pathway, cAMP-signaling pathway, MAPK signaling pathway, aldosterone synthesis, and secretion, and thyroid hormone synthesis. Lysine degradation was found to be the most significant pathway underlying LFE group. Additionally, the over-represented pathways included arginine and proline metabolism, and fatty acid biosynthesis as well. Pathways like fatty acid elongation and degradation, valine, leucine, and isoleucine degradation, lysine degradation, and tryptophan metabolism were identified for the LHFE group (**Figure 7**).

Gene annotation, reference SNP ID, *Sus scrofa* EnsemblID, chromosome in which the SNPs are located, and their connectivity scores as calculated for each breed and the LFE and HFE groups are given in **Supplementary Table 3**. A higher value of connectivity scores (in hundreds or above) shows that the data exhibit a strong driver that makes the subset of the samples globally different from the rest.

## Discussion

Feed efficiency in pigs is an important quantitative trait influenced by complex genetic control. Understanding the molecular mechanisms underlying this complex trait will help in the efficient selection of pigs, thereby beneficial for the pig producers. In Danish pig breeding, Duroc is currently used as terminal sires in combination with crossbred Landrace x Yorkshire families (Do et al., 2013). Selection emphasis on feed efficiency in Duroc is greater as compared to Landrace. In our study, we found that Landrace has significantly greater FCR as compared with Duroc, this was also consistent with the studies reported earlier (Jensen et al., 2007; Do et al., 2013).

A plethora of GWAS and QTL studies for feed efficiency in pigs have been reported. However, to our knowledge, genetic networks based on HT SNP data for FE using genome-wide epistatic interactions have not been reported in Duroc and Landrace pigs. In this study, we used genome-wide SNPs to unravel the genetic contribution of the epistatic effects to the phenotypic variation for feed efficiency related traits like FCR and RFI in pigs. For this purpose, we used high throughput SNP genotype data generated with GGP Porcine HD array to search for signature SNPs that highly interact with each other, followed by network and pathway over-representation analyses, thereby underlying FE related-traits in Danish production pigs.

The pair-wise interaction varied between both the breeds and within the low and high feed efficient groups in each breed. While this does not give an accurate representation of individual interactions, it does indicate which chromosomes may be hot spots for interactions of a given phenotype. From the network analysis, we found several modules associated with FCR and RFI in both breeds, as well as in low and high feed efficient groups of each breed pointing towards the pathways affecting these traits.

Based on the over-representation pathway analysis, *Rap1* and *Ras* signaling pathway, inositol phosphate metabolism, ErbB signaling pathway, thyroid hormone synthesis, adipocytokine-signaling pathway were identified in Duroc. From the SNP-SNP interaction in DLFE, thiamine metabolism, folate biosynthesis, and phenylalanine, tyrosine and tryptophan metabolism were found to be involved. In DHFE group, the pathways resulting from the interaction were hippo signaling pathway, glycosaminoglycan biosynthesis, glycosylphosphatidylinositol (GPI)-anchor biosynthesis, and nitrogen metabolism. In Landrace, the major pathways involved were cGMP-PKG signaling pathway, cAMP-signaling pathway, MAPK signaling pathway, aldosterone synthesis and secretion, and thyroid hormone synthesis. Furthermore, lysine degradation was found to be the most significant pathway underlying LLFE group, followed by arginine and proline metabolism and fatty acid biosynthesis as some other pathways. Pathways like fatty acid elongation and degradation, valine, leucine and isoleucine degradation, lysine degradation, and tryptophan metabolism were identified for the LHFE group.

### mTOR and MAPK: Key Signaling Pathways in Duroc

The top pathways over-represented in Duroc included the ErbB signaling pathway, Ras, and Rap1 pathways. ErbB signaling pathway is associated with the *NRG4* gene as given in our study. The ErbB receptor tyrosine kinases include the epidermal growth factor receptor (EGFR), ErbB2, ErbB3, and ErbB4. These are important for intestinal tract homeostatic maintenance (Schumacher et al., 2017). They have shown the treatment of activated pro-inflammatory macrophages with the ErbB4 ligand neuregulin-4 (*NRG4*) induces apoptosis (Schumacher et al., 2017). One of the major downstream targets of ErbB tyrosine receptor family is P13-K/Akt (Schuurbiers et al., 2009). Activation of this pathway is achieved after activation of EGFR and four members of ErbB family. EGFR itself is a weak activator of P13-K, but connects to Ras/P13-K/Akt pathway or collaborates with ErbB3 (Schuurbiers et al., 2009). Ras along with Rap1 play a critical role in regulating T-cell proliferation response (Remans et al., 2004). Ras transmits signals from TCR to the activation of Raf-1/ERK signaling cascade required for T-cell proliferation, IL-2 production, and thymic maturation. Closely related, Rap1 stimulates TCR and suppresses Ras-dependent transformation (Remans et al., 2004). P13Ks phosphorylate 3′-hydroxyl group of the inositol ring of phosphatidylinositides and are divided into three classes: Class I P13K, PIP3, and PIP2. PIP3 acts as a secondary messenger, facilitating the recruitment and activation of PI3K-dependent kinase-1 (*PDK1*) (Yu and Cui, 2016). The signaling duration of PIP3 is subject to regulation by phosphatase and tensin homolog (*PTEN*), which acts to oppose PI3K activity. The serine/threonine kinase AKT, also known as protein kinase B (*PKB*), possesses a PH domain and is recruited to the plasma membrane along with PDK1. Phosphorylation of amino acid residues by *PDK1* and mTORC2 (a subunit of mTOR from P13K-related kinase family), respectively, is essential for full AKT activation (Yu and Cui, 2016). Previous reports of *AKT3*, which is a key regulator of P13/Akt/mTOR pathway, is involved in ErbB, Ras, Rap1, cGMP-PKG pathways in our study. Horodyska et al. (2019) also reported Akt acting in hepatocyte growth factor (HGF) signaling and epidermal growth factor (EGF) signaling pathways. Akt is a member of the Akt kinase family and has a significant role in the modulation of cell survival and proliferation in high feed efficient pigs (Horodyska et al., 2019). Gene *ADCY8*, which was involved in most of the pathways in our study, was involved with cholesterol levels in pigs (Bovo et al., 2019).

Genes *ABL1*, *MAP3K4*, *MAP3K5* (chr 1), *SEMA6A* (chr 2), *KITLG* (chr 5), and *KAT2B* (chr 13), identified from highly interconnected epistatic SNPs also pointed out towards ErbB, Ras, Rap1, thyroid hormone, axon guidance signaling pathways in Duroc. Mitogen-activated protein kinase (MAPK) cascades have been shown to play a key role in transduction extracellular signals to cellular responses (Zhang and Liu, 2002), and its overexpression increased axon branching (Kalil et al., 2011). The top connectivity score SNPs pointed towards genes like *SEMA6A*, *ABLIM1*, and *NTNG1*, which are involved in the axon guidance pathway. *SEMA6A* was downregulated in porcine skeletal muscle as reported in a study of the transcriptional response to feeding a linseed enriched diet in pigs (Wei et al., 2016). *ABLIM1* and *NTNG1* affect intramuscular fat content (IMF) (Wang et al., 2017; Wang et al., 2019). Some other high connectivity score SNPs pointed to the *MAP3K5* gene, which was related to residual feed intake (Pu et al., 2016). The MAPK (ERK1/2) signaling pathway causes serine phosphorylation by MAPK of several nucleoproteins, including the nuclear thyroid hormone receptor beta1, which is activated by thyroid hormone (Tang et al., 2004)*. KAT2B* was involved with thyroid hormone signaling pathway. The systemic effects mediated by thyroid hormones induces metabolic shifts characterized by increased lipolysis and gluconeogenesis, affecting feed efficiency in pigs (Reyer et al., 2017a). MAPKs regulates (either stimulating or inhibiting) the catalytic activity, and specificity, of kinases and phosphatases that are involved in the metabolism of phosphatidylinositols (PI) and inositol phosphates (IP), thereby exert regulatory actions on PI- and/or IP-dependent signaling pathways (Caldwell et al., 2006). Genes *P14KB* and *PLCB1* which accelerated to Inositol phosphate metabolism were associated with phosphate metabolism and feed efficiency in Duroc (Ding et al., 2018). *P14KB* was reported to be enriched in phosphate metabolism in Berkshire and Korean pigs (Edea and Kim, 2014).

We also focused on the study of the pathways identified by the highly interacting SNPs in LFE and HFE groups of both the breeds. In DLFE group, *TPK1* (chr 9) was significantly involved with thiamine metabolism. The most active form of vitamin B1 is thiamin pyrophosphate; its synthesis in eukaryotes requires thiamine pyrophosphokinase, which catalyzes pyrophosphate group transfer from ATP to thiamine (Tylicki et al., 2018). Thiamin supplementation in dairy cattle has been reported to increase rumen pH and balance the population of lactic acid-producing and -consuming bacteria (Wang et al., 2015). Thiamine is critical for cellular function, as its phosphorylated and active form, thiamine diphosphate (TDP), acts as a coenzyme for three key enzymes in glucose metabolism (Liu et al., 2017) which differs to a great extent in high and low feed efficient groups (Fu et al., 2017).

From DHFE, the genes *CRB1* (chr 10), *DLG2* (chr 9), *PARD6G* (chr 6), and *PPP1CB* (chr 3) pointed towards the hippo signaling pathway. This pathway is an evolutionarily conserved and it controls organ size by regulating cell proliferation, apoptosis, and stem cell self-renewal (Leno-colorado et al., 2017). The glycosaminoglycan pathway, involving the *CHST4* (chr 6) gene, was also significant in the HFE group. This pathway was identified in a GWAS study of beef cattle for growth and intake components for feed efficiency (Serão et al., 2013).

### Cross-Talk of cGMP and cAMP Pathway Regulating Aldosterone Secretion in Landrace

The top pathways in Landrace were endocytosis and signaling pathways including cGMP-PKG, cAMP, estrogen signaling pathways, adrenergic signaling in cardiomyocytes, insulin secretion, thyroid hormone synthesis, aldosterone synthesis, amyotrophic lateral sclerosis, and purine metabolism. Cyclic nucleotides 3′,5′-cyclic adenosine monophosphate (cAMP) and 3′,5′-cyclic guanosine monophosphate (cGMP) are ubiquitous intracellular second messengers that regulate multiple physiological functions (Pavlaki and Nikolaev, 2018). The high interacting epistatic SNPs point out towards genes *GABBR2*, *GNA12, PRKCG* from chromosome 1, 3, and 6, which underlie the thyroid hormone, cGMP-PKG, and cAMP pathways. Phosphodiesterases (PDEs) are hydrolyzing enzymes that terminate the intracellular effects of cyclic nucleotides by their hydrolysis and prevents continuous activation of the downstream effector proteins (Pavlaki and Nikolaev, 2018). cGMP-regulated PDEs, especially *PDE2* are found to regulate cGMP-to-cAMP cross-talk (Pavlaki and Nikolaev, 2018). Aldosterone secretion requires PDE2A-mediated hydrolysis of cAMP (Pavlaki and Nikolaev, 2018). The *ATP1A2* gene, acting in most of the pathways as mentioned above, was involved with production traits in pigs (Stefanon et al., 2004) and was also related to cGMP-PKG signaling pathway in our study. Adrenergic signaling in cardiomyocytes was over-represented here, where *CACNG7* and *CACNG5* were partaking. According to the studies reported earlier, *CACNG7* is a candidate gene with feed efficiency in Nelore cattle (Olivieri et al., 2016) while *CACNG5* was differentially expressed and participated in activating MAPK signaling pathway in chickens (Li et al., 2018). *MAPK* pathway is activated by several stimuli and transduce the signal inside cells, generating diverse responses, including cell proliferation, differentiation in the rapidly renewing epithelia that line the gastrointestinal tract (Osaki and Gama, 2013).

In Landrace, lysine degradation was involved with LFE group. Fatty acid elongation, synthesis, and degradation of ketone bodies, valine, leucine, and isoleucine degradation, lysine degradation, metabolism of tryptophan, pyruvate, propanoate, butanoate, glyoxylate, and dicarboxylate, ferroptosis and cell adhesion molecules pathways were found over-represented in HFE. Genes *SETD7* (chr 8) and *PLOD1* (chr 6) were involved with lysine degradation in our study. Lysine is a limiting amino acid, and its deficiency impairs the animal’s immunity and growth performance (Liao et al., 2015). Reports also suggested that the dietary supplementation with lysine influences intestinal absorption and metabolism of amino acids (Yin et al., 2017). Lysine restriction inhibits intestinal lysine transport and promotes feed intake associated with gut microbiome in piglets (Yin et al., 2017). From the LHFE group*, ACAT1* (chr 9) was involved in most of the pathways, which are also found to affect lipid metabolism in feed efficiency in pigs (Reyer et al., 2017a). Gene *LPCAT3* involved with ferroptosis incorporates arachidonic acid (lipid) into the membrane of the intestine and liver cells, which enables triacylglycerols to be assembled into lipoproteins (Hashidate-Yoshida et al., 2015). Triglycerides are found to affect energy value to animals. Major constituents of triacylglycerol, saturated fatty acid (SFA) and monounsaturated fatty acid (MUFA) (Kasprzyk et al., 2015) were found to be lower in high feed efficient pigs (Horodyska et al., 2019).

The annotated SNPs and genes underlying the significant over-represented pathways discussed here pointed out their role in feed efficiency related-traits. We also identified other SNPs with high SNP-SNP interaction values, high connectivity score, and clustered into the selected modules. However, they were in intergenic regions and thus were not included in gene and pathway analysis. Since these SNPs occur in the modules selected to have a high epistatic interaction among themselves, a detailed study of these SNPs is needed to understand their role in feed efficiency related-traits.

## Conclusion

This study applied a novel approach for studying the genome-wide epistatic interaction in quantitative traits using high throughput genomic data under a network and systems biology context. To the best of our knowledge, it is the first study to report such genome-wide genetic interaction networks underlying feed efficiency traits in pigs. We used WISH-R and other network approaches to search for pair-wise SNP interaction for feed efficiency traits from genotype data based on prior biological knowledge and frameworks. Highly interacting epistatic SNPs that clustered together in significant modules (r > 0.4, p ≤ 0.05), were identified in Duroc and Landrace across the whole population for FCR and RFI traits. The approach followed here, provided many interesting genes and interactions with significant p-values. While some of the identified hub-SNPs were linked to genes well known for their association with feed efficiency, others are novel and potentially provide new avenues for further research. The main over-represented pathways in Duroc were the mTOR and MAPK pathways. In Landrace, the main pathways were cAMP, cGMP-PKG pathway, aldosterone synthesis and secretion, and purine metabolism. Further validation of epistatic SNPs and genes identified here in a larger cohort would help to establish a framework for modelling epistatic variance in future methods of genomic prediction that includes epistasis, increasing the accuracy of estimated genetic merit for FE, as well as helping the pig breeding industry.

## Data Availability Statement

We have made our genotype data along with metadata publicly available in NCBI-GEO data repository with the accession number: GSE144064 and the link: https://www.ncbi.nlm.nih.gov/geo/query/acc.cgi?acc=GSE144064.

## Ethics Statement

The feed efficiency experiment were approved and carried out in accordance with the Ministry of Environment and Food of Denmark, Animal Experiments Inspectorate under the license number (tilladelsesnummer) 2016-15-0201-01123, and C-permit granted to the principal investigator/senior author (HK).

## Author Contributions

HK conceived and designed this “FeedOMICS” project, obtained funding as the main applicant. VC and HK designed the blood sampling experiments, phenotype data collection, and biostatistical/bioinformatics analyses. PB and VC carried out biostatistical and bioinformatic data analyses. All authors collaborated in the interpretation of results, discussion, and write up of the manuscript. All authors have read, reviewed, and approved the final manuscript.

## Funding

This study was funded by the Independent Research Fund Denmark (DFF) – Technology and Production (FTP) grant (grant number: 4184-00268B).

## Conflict of Interest

The authors declare that the research was conducted in the absence of any commercial or financial relationships that could be construed as a potential conflict of interest.

## Abbreviations

Chr, Chromosome; DFI, Daily feed intake; DHFE, Duroc high feed efficient; DLFE, Duroc low feed efficient; EDTA, Ethylenediaminetetraacetic acid; FC, Feed consumed; FCR, Feed conversion ratio; FE, Feed efficiency; GWAS, Genome-wide association study; HTG, High throughput data; KEGG, Kyoto Encyclopedia of Genes and Genomes; LD, Linkage disequilibrium; LHFE, Landrace high feed efficient; LLFE, Landrace low feed efficient; pDFI, Predicted daily feed intake; RFI, Residual feed intake; SNP, Single nucleotide polymorphism; TOM, Topological overlap matrix; WGCNA, Weighted gene Co-expression network analysis; WISH, Weighted Interaction SNP hub.
